# Rare Variant Analysis and Molecular Dynamics Simulation in Alzheimer’s Disease Identifies Exonic Variants in FLG

**DOI:** 10.3390/genes13050838

**Published:** 2022-05-07

**Authors:** Weixue Xiong, Jiahui Cai, Ruijia Li, Canhong Wen, Haizhu Tan, on behalf of the Alzheimer’s Disease Neuroimaging Initiative (ADNI) Database

**Affiliations:** 1 Department of Preventive Medicine, Shantou University Medical College, Shantou 515000, China; 20wxxiong@stu.edu.cn (W.X.); 19jhcai@stu.edu.cn (J.C.); 2 Department of Statistics and Finance, School of Management, University of Science and Technology of China, Hefei 230000, China; euruijia@outlook.com

**Keywords:** Alzheimer’s disease, rare variants, ADNI, MIRAGE

## Abstract

Background: Although an increasing number of common variants contributing to Alzheimer’s disease (AD) are uncovered by genome-wide association studies, they can only explain less than half of the heritability of AD. Rare variant association studies (RVAS) has become an increasingly important area to explain the risk or trait variability of AD. Method: To investigate the potential rare variants that cause AD, we screened 70,209 rare variants from two cohorts of a 175 AD cohort and a 214 cognitively normal cohort from the Alzheimer’s Disease Neuroimaging Initiative database. MIRARE, a novel RVAS method, was performed on 232 non-synonymous variants selected by ANNOVAR annotation. Molecular docking and molecular dynamics (MD) simulation were adopted to verify the interaction between the chosen functional variants and BACE1. Results: MIRAGE analysis revealed significant associations between AD and six potential pathogenic genes, including *PREX2*, *FLG*, *DHX16*, *NID2*, *ZnF585B* and *ZnF875*. Only interactions between FLG (including wild type and rs3120654(SER742TYR)) and BACE1 were verified by molecular docking and MD simulation. The interaction of FLG(SER742TYR) with BACE1 was greater than that of wildtype FLG with BACE1. Conclusions: According to the literature search, bio-informatics analysis, and molecular docking and MD simulation, we find non-synonymous rare variants in six genes, especially FLG(rs3120654), that may play key roles in AD.

## 1. Introduction

Alzheimer’s disease (AD) is a devastating illness that always causes progressive neurodegenerative and cognitive decline [1]. According to the report from WHO (key facts of dementia. Available online: https://www.who.int/news-room/fact-sheets/detail/dementia (accessed on 2 September 2021)) [2], there were approximately 35 million AD patients worldwide in 2021. The heritability of AD is high (79%) and more than 50% of the phenotypic variance can be explained by genetic variations [3,4]. An increasing number of common variants (minor allele frequency (MAF) > 0.05) that cause AD has been uncovered by large-scale genome-wide association studies. For example, *RBFOX1* with a higher amyloid-β burden has been linked to early AD [5]. Apolipoprotein E-4 (*APOE4*), an important possible AD therapeutic target, is highly expressed in more than half of AD patients [6]. However, these common variants explain less than half of the heritability to date [7,8]. For example, the total proportion of heritability of *APOE4* is estimated to be 25% [9,10]. With the development of base-by-base resolution of whole-genome sequencing, low-frequency variants (0.01 < MAF < 0.05) and rare variants (0.005 < MAF < 0.01), previously difficult to detect by microarrays, are becoming attractive alternative biomarks to AD [11,12,13]. The reason might be that these two types of variants can explain additional disease or trait variability because the growth of the effect sizes of associated variants along with the drop of allele frequencies can exceed the limits imposed by natural selection on more common variants [11]. For example, rare variants in *TREM2* have been reported to be associated with a significant increase in the risk of AD [14], and rare variants in three genes (*APP*, *PSEN1* & *PSEN2*) can explain 5–10% of the occurrence of early onset AD [15,16]. Therefore, the identification of rare variants associated with AD is becoming increasingly important.

Due to the importance of rare variants in sequencing studies, rare variant association studies (RVAS) have become an increasingly important area to explain additional disease risk or trait variability [17,18]. Current approaches for RVAS in complex traits involve burden tests, sequence kernel association tests (SKAT), variable threshold (VT), MIxture model based Rare variant Analysis on GEnes (MIRAGE), etc. In order to determine causative gene variants, MIRAGE, a novel method for determining causative gene variants, was applied in our study because it is an effective method to analyze rare variant data from case–control studies. MIRAGE uses an empirical Bayesian approach to estimate these prior probabilities by combining information across genes [19]. It not only better accounts for the heterogeneity of variant effects of genes, but also selects putative causal variants by fully using external information [19]. 

After selecting the significant variants for AD by using the RVAS method, it is very important to verify their relevant functions and mechanisms for AD. Over 80% of gene substrates (proteins) rarely act alone, but in complexes [20]. Protein–protein interactions (PPIs) are a useful way to predict the functionality and mechanism of a potential protein by acting with a known protein to disease [21] because proteins involved in the same cellular processes always interact with each other [21]. In an abundance of studies, amyloid β (Aβ) accumulation occurs before clinical symptoms in the early progression of AD [22,23]. Aβ is generated by β-site APP-cleaving enzyme 1 (BACE1) by catalyzing the amyloid precursor protein (APP), and inhibition of BACE1 activity can prevent AD development [22,23,24,25]. However, natural and synthetic BACE1 inhibitors have consistently failed in the treatment of AD for reasons such as liver toxicity and lack of improvement in cognitive decline [26]. Further exploration of the pathology of BACE1 in AD is needed. Hence, BACE1 is chosen as the interaction party by several while investigating PPIs between BACE1 and the potential proteins of AD by using molecular docking, molecular dynamics (MD) simulation, etc. 

The aim of our study is to explore the genetic architecture of AD. Genetic data in our study are obtained from the Alzheimer’s Disease Neuroimaging Initiative (ADNI) database (http://adni.loni.usc.edu, accessed on 9 September 2020) [27]. To efficiently boost the power of the RVAS and genomic evaluation accuracy, whole-genotype imputation on those preprocessed genomes was first implemented on the Michigan imputation server (https://imputationserver.sph.umich.edu, accessed on 25 November 2020). Then, imputed variants were functionally annotated and classified as downstream, exonic, intergenic, intronic, ncRNA-exonic, ncRNA-intronic, ncRNA splicing, splicing, and upstream by using ANNOVAR (modified on 7 October 2020) [28], using several databases, including RefGene, exac03, avsnp147, dbnsfp30a, and ljb26_all. The MIRAGE method was subsequently adopted to perform RVAS analysis [19]. After that, several bio-informatics tools further provided a comprehensive demonstration of the significant selected variants. In addition, homology modeling, molecular docking, and MD simulation were performed to find the PPIs between the selected genes and BACE1 [29], and the effects of mutations on them.

## 2. Materials and Methods

**Figure 1:**
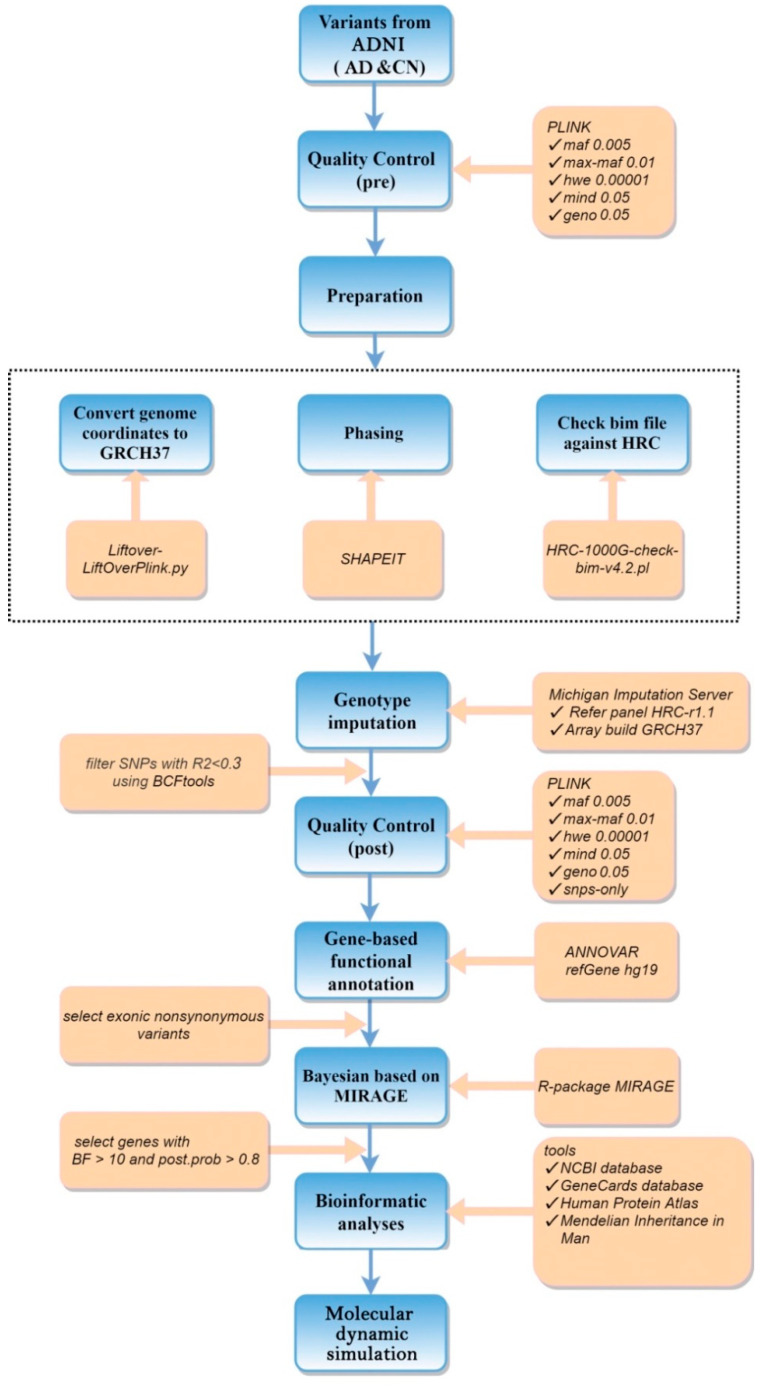
Rare variant association studies workflow.

Detailed RVAS workflow is provided in Figure 1.

### 2.1. Participants 

Data in this study was downloaded from the ADNI-1 database, including 600,470 genetic data, several demographic and clinical data (such as age, gender, and race). Overall, 175 AD and 214 cognitively normal (CN) subjects, aged from 56.4 to 90.9 years, were enrolled. Written informed consent was obtained from all participants. The full list of inclusion and exclusion criteria can be accessed online at https://adni.loni.usc.edu/wp-content/uploads/2008/07/adni2-procedures-manual.pdf, accessed on 3 May 2022.

### 2.2. Statistical Analysis

#### 2.2.1. Demographic Comparisons

The two-tailed student’s *t*-test for two cohort comparisons in age was performed. The chi-squared test was adopted to determine the differences in gender and race distribution between two groups. 

#### 2.2.2. Data Preprocessing

A series of steps, including quality control (QC) before genotype imputation like filtering for single nucleotide polymorphism (SNPs) with call rate (missing > 5%), MAF > 1% and MAF < 0.5%), and the Hardy–Weinberg equilibrium (*p*-value < 1 × 10^−5^), LiftOver for converting the genome coordinates into hg19, SHAPEIT2 for phasing and whole-genotype imputation on the Michigan imputation server (https://imputationserver.sph.umich.edu), were carried out. To filter imputed data with high degrees of uncertainty, an R-square of 0.3 was set for inclusion after imputation. Then, post-imputation QC and gene-based annotation were implemented. In this study, non-synonymous exonic variants occurring in 330 subjects were collected from the annotated variants because non-synonymous mutations were demonstrated to have a much greater effect on an individual than synonymous mutations [30]. Non-synonymous variants were subsequently predicted to be at risk or non-risk variants by considering three functionally damaging effect scores, such as polyPhen2 HDIV scores [31], Sorting Intolerant From Tolerant (SIFT) scores [32] and Combined Annotation-Dependent Depletion (CADD) scores [33] together. 

#### 2.2.3. RVAS Analysis

MIRAGE was conducted to do RVAS analysis on whole exome sequencing data of 330 subjects. Variant groups are defined depending on their annotations. Those variants with 64 PolyPhen scores lower than 0.1, a CADD score 10 or SIFT score < 0.05 were labeled as group1; others were labeled group 2. The number of the case and control groups was equal to the total number of alleles in cases and controls at a genomic locus. γ was set to 2. With the estimated parameters, the Bayes factor (BF) and posterior probabilities of each gene were computed to assess its association with the disease by comparing the likelihood of the full model (group 1) to the likelihood of the null model (group 2). In this study, a threshold of BF > 3 was set to select rare potential risk variants associated with AD.

### 2.3. Bioinformatic Analyses 

Several in silico prediction tools were used to verify the underlying biological interpretations of the identified SNPs based on bio-informatics databases and resources, including NCBI (https://www.ncbi.nlm.nih.gov/gene/, accessed on 3 May 2022), GeneCards (https://www.genecards.org/, accessed on 3 May 2022), UCSC Genome Browser on Human (https://genome.ucsc.edu/, accessed on 3 May 2022), and Online Mendelian Inheritance in Man (https://omim.org/entry/, accessed on 3 May 2022). Online tools for the effect of SNPs on protein structure and function were also used, such as: polyPhen2 (http://genetics.bwh.harvard.edu/pph2/index.shtml, accessed on 3 May 2022), SIFT (http://provean.jcvi.org/index.php, accessed on 3 May 2022), and CADD (https://cadd.gs.washington.edu/snv, accessed on 3 May 2022).

### 2.4. Molecular Dynamics Simulation

To demonstrate whether the proteins transcribed by potential pathogenic genes were involved in the pathogenic mechanism of AD or not, MD simulations were used to verify the stability of the complex docked with BACE1, which is involved in the formation of Aβ. All of the proteins that were finally chosen by MIRAGE were modeled on the trRosseta server, and the point amino acid was mutated by Chimera 1.14 [34,35,36]. The quality of protein structure was evaluated by PROCHECK [37]. Each selected protein was separately docked with BACE1 by the High Ambiguity Driven protein-protein Docking (HADDOCK) [38]. Finally, MD simulation was performed to test whether the above genes were involved in the formation of Aβ or not, and the influence of non-synonymous mutations on them.

## 3. Results

### 3.1. Demographic Comparisons

Table 1 describes the results of the difference tests between the AD and CN groups. From Table 1, we find that there were no significant differences in age (*p*-value = 0.907), sex (*p*-value = 0.815) and race (*p*-value = 0.357) between the two groups.

### 3.2. RVAS Analysis

After whole-genotype imputation and QC steps, a total of 330 participants with 70,209 variants passing were reserved.

The predicted model using a data set that includes 232 non-synonymous variants in 150 ADs and 180 NCs was built by MIRAGE. Table 2 shows that two non-synonymous variants (*FLG* (BF = 4040.782) and *ZNF585B* (BF = 87.062)) are likely to be pathogenic when considering BF and post-probability together.

### 3.3. Molecular Dynamics Simulation

**Figure 2:**
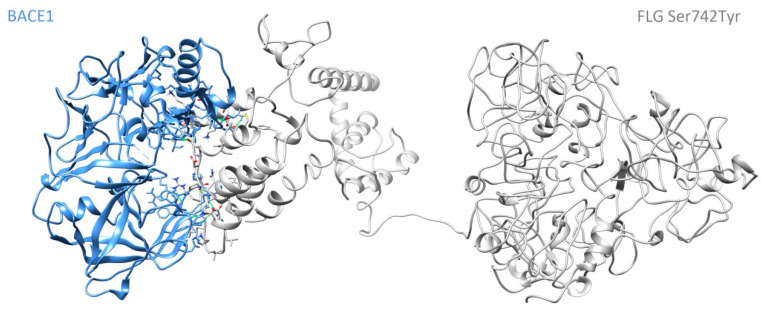
Three-dimensional protein structure of the complex BACE1-FLG (Ser742Tyr).

The interaction strength of the protein complex that binds is denoted by the overall HADDOCK score, which is composed of the weighted average of the Van der Waal’s interactions, electrostatic energy, desolvation energy, and the buried surface area (BSA). The lower the HADDOCK score is, the stronger the interaction will be. As Table 3 shows, the interaction between BACE1- mutant FLG (rs3120654 (Ser742Tyr)) is stronger than the interaction between BACE1-FLG because the HADDOCK score of BACE1-FLG (Ser742Tyr) is lower than that of BACE1-FLG. Figure 2 shows the 3D protein structure of the complex BACE1-FLG (Ser742Tyr). There was no interaction between ZNF585B and BACE1. 

**Figure 3:**
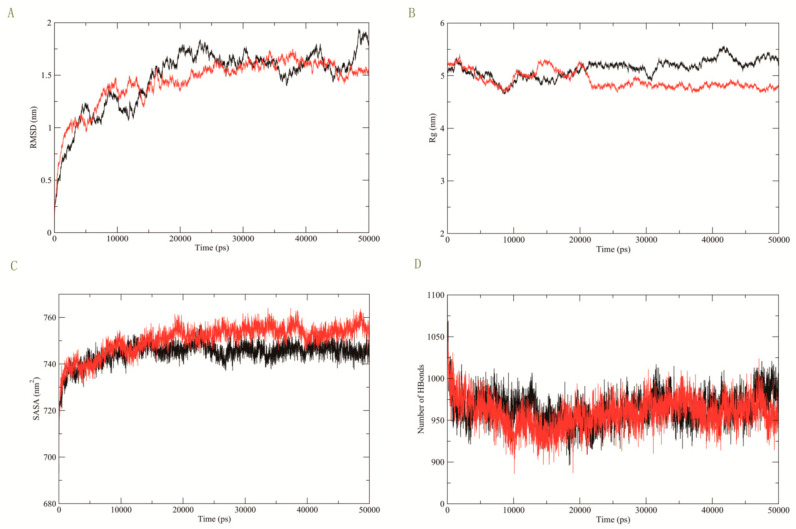
MD simulation results of BACE1-FLG and BACE1-FLG (Ser742Tyr) mutant. (**A**) The RMSD plot depicts that there are no erratic fluctuations in the molecular systems and all complexes are stable. (**B**) The result of the Rg elucidates the volumetric and compactness variations induced in the complex. (**C**) The result of SASA for the protein structures with dimensional discrepancy. (**D**) The hydrogen bonds reflect the protein’s rigidity and its ability to interact with its partners.

The MD simulation results are shown in Figure 3 below. The root-mean-square deviation (RMSD) plot depicts that there are no erratic fluctuations in the molecular systems and all complexes are stable (see Figure 3A). The result of the radius of gyration (Rg) in Figure 3B elucidates the volumetric and compactness variations induced in the complex. Figure 3C shows the results of solvent-accessible surface area (SASA) for the protein structures with dimensional discrepancy. From Figure 3D, we can also find that the hydrogen bonds reflect the protein’s rigidity and its ability to interact with its partners (Color scheme: black: BACE1-FLG, red: BACE1-FLG (Ser742Tyr) mutant).

## 4. Discussion

The results of MIRAGE showed that *PREX2*, *FLG*, *DHX16*, *NID2*, *ZnF585B,* and *ZnF875* have pathogenic risk rare variants for AD. *PREX2* is associated with brain arteriovenous malformations that can induce vascular amyloid β deposition, which is a significant risk factor for AD [39,40,41]. Elena Galea et al. also suggest that *PREX2* is one of the top genes detected by principal component analysis when they define AD astrocytes in multi-transcriptomic analysis [42]. The RNA helicase *Dhx16* induces transcription alterations and DNA methylation changes that are involved in memory-related neurological and neuropsychiatric diseases [43]. In single variant analysis, candidate genes including *DHX16* were identified as novel candidates for early onset AD in the study by Victoria Fernandez et al. [44]. Nidogen 2 (*NID2*) has been discovered to cause neurological disease [45]. *NID2* is a pathogenic gene for neurological disease because the severity of cerebral amyloid angiopathy is least in the striatum, where *NID2* is reduced [46]. *ZnF875* (also known as *HKR1*) is associated with aging [47]. Miren Altuna’s studies find that the methylation level of *HKR1* is significantly correlated with the burden of phosphorylated tau deposits [48]. The relationship between *ZNF585B* or *ZnF875* and AD is still unknown. 

Since BF greater than 10 is considered strong evidence, while a posterior probability greater than 0.8 indicates a potential efficacy signal. *FLG* (BF = 4040.782, posterior probability = 0.996) and *ZNF585B* (BF = 87.062, posterior probability = 0.842) are considered as the most likely candidate genes for rare variants [49,50]. *ZNF585B* is a P53 inhibitor, and P53 is up-regulated in AD [51,52]. *ZNF585B* is up-regulated in the olfactory bulb neural stem (OBNS) as a modulated gene, whereas OBNS enriches the ErbB signaling pathway [53]. Lack of ErbB signaling in humans has been implicated in the development of neurodegenerative diseases, such as AD [53]. 

In our study, filaggrin (*FLG*) was the only one of these genes with rare variants involved in the BACE1 pathway by molecular docking and MD simulation. The protein encoded by *FLG* is an intermediate filament-associated protein that aggregates keratin intermediate filaments in mammalian epidermis [54]. Shafiq M et al. suggested that significantly higher levels of *FLG* are observed in rapidly progressive Alzheimer’s disease high density factions (HDFs) compared with sporadic Creutzfeldt-Jakob disease-specific HDFs [55]. Our bioinformatics analysis identified that three Reactome pathways for the *FLG* gene are related to developmental biology and include nervous system development (https://reactome.org/PathwayBrowser/#/R-HSA-1266738, accessed on 3 May 2022), formation of the cornified envelope, which is involved in axonal regeneration [56], and keratinization (Keratin 9 has been identified as an important biomarker for AD [57]). 

**Figure 4:**
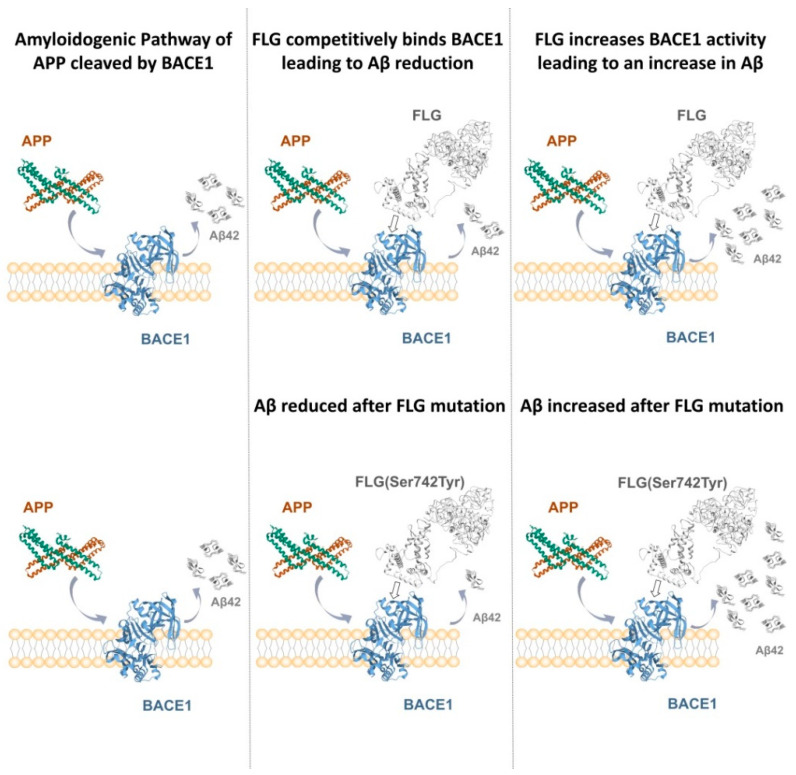
Hypothetical theoretical pathway figure of FLG and its mutant FLG (Ser742Tyr) on Aβ42 accumulation.

The validation of interactions, using 50 ns (50,000 ps) MD simulations, between FLG and BACE1 showed stable binding between BACE1 and FLG. This suggests that FLG may affect the formation of Aβ by affecting the activity of BACE1, and thus participate in the pathogenesis of AD. If FLG was competitively bound to BACE1 or the interaction could lead to a decrease in BACE1 activity, then the competitive binding of APP to BACE1 would be decreased, leading to a reduction in Aβ42 produced by hydrolysis, which can alleviate the symptoms of AD. If the interaction between FLG and BACE1 could increase the activity of BACE1, the activity of BACE1 would be increased, leading to an increase in Aβ42 produced by hydrolysis, promoting the development of AD symptoms. Additionally, mutant FLG (Ser742Tyr) can affect the interaction with BACE1. These results show that the interaction between FLG and BACE1 is enhanced by the FLG (Ser742Tyr) mutation, which further supports the effect of the rare variants we found related to AD. The theoretical pathway figure is as shown in Figure 4 below.

Thus far, a large amount of literature, the results of bioinformatics analysis and the results of MD simulation support the existence of pathogenic rare variants in these selected genes. Only BACE1 was introduced to interact with each significant gene in our docking and MD experiment in this study. Hence, in the future, many known proteins related with AD will be introduced to do the validation, and experiments in vitro and vivo will be performed to assess the computational work. In addition, the sample size in our study is not large enough for our RVAS research. 

In conclusion, we identified six genes with rare variants affecting AD, especially *FLG*. We hope our study may aid in furthering study of exploring the underlying pathology mechanism of AD.

## Figures and Tables

**Table 1:** Comparisons of demographics and clinical variables between AD and CN cohorts.

		AD	CN	*p*-Value
No. of participants, n (%)		175 (45%)	214 (55%)	
Sex, n (%)	M	93 (53%)	115 (54%)	0.907
	F	82 (47%)	99 (46%)	
Age, Median (IQR)		75.8	75.5	0.815
		(70.85, 81.10)	(72.12, 78.38)	
Race, n (%)	Asian	2 (1)	2 (1)	0.357
	Black	8 (5)	15 (7)	
	More than one	2 (1)	0 (0)	
	White	163 (93)	197 (92)	
Education, Median (IQR)		15 (12, 16)	16 (14, 18)	<0.001
Participant residence, n (%)	House	121 (69)	162 (76)	0.005
	Condo/Co-op (owned)	17 (10)	32 (15)	
	Apartment (rented)	21 (12)	13 (6)	
	Mobile Home	3 (2)	2 (1)	
	Retirement Community	5 (3)	3 (1)	
	Assisted Living	7 (4)	0 (0)	
	Other	1 (1)	2 (1)	
*APOE4*, n (%)	0	58 (33)	156 (73)	<0.001
	1	85 (49)	53 (25)	
	2	32 (18)	5 (2)	

**Table 2:** The results of RAVIS analysis by using MIRAGE.

Gene	BF	Post.Prob
*FLG*	4040.782	0.996
*ZNF585B*	87.062	0.842
*ZNF875*	9.331	0.364
*PREX2*	6.746	0.293
*NID2*	5.819	0.263
*DHX16*	4.515	0.217

**Table 3:** Docking results of BACE1 interaction with selected proteins.

	BACE1-FLG	BACE1-FLG Ser742Tyr
HADDOCK score	−77.500 ± 13.900	−89.600 ± 16.000
Root Mean Square Deviation from the overall lowest-energy structure	0.500 ± 0.300	0.800 ± 0.500
Van der Waals energy	−79.400 ± 5.400	−83.800 ± 14.200
Electrostatic energy	−516.500 ± 81.300	−566.100 ± 71.800
Desolvation energy	2.400 ± 7.700	2.700 ± 3.400
Buried Surface Area	3250.100 ± 135.700	3318.500 ± 346.700
Z-Score	−1.400	−1.800

Notes: (1) BACE1-FLG refers to docking BACE1 with FLG; (2) BACE1-FLG (Ser742Tyr) refers to docking BACE1 with the FLG (Ser742Tyr) mutant; (3) HADDOCK score shows how well the protein complex binds with each other and is obtained from the weighted average of the Van der Waals interactions, electrostatic energy, desolvation energy, and the buried surface area.

## Data Availability

Data in our study are obtained from the Alzheimer’s Disease Neuroimaging Initiative (ADNI) database (http://adni.loni.usc.edu, accessed on 3 May 2022), an open source database.
